# Supplementary material for: What does it take to learn the rules of RNA base pairing? A lot less than you may think
Source: bioRxiv. 2025 Aug 2:2025.07.31.668042. Preprint. [Version 1] doi: 10.1101/2025.07.31.668042 (PMC12324431; doi:10.1101/2025.07.31.668042)
Supplement: Supplement 1 [file media-1.gz › supplemental_material/src/rscape_v2.6.0.tar.gz!/rscape_v2.6.0/lib/infernal/Manuscripts/banded-cyk/figs/cmintro_bandcyk.pdf]

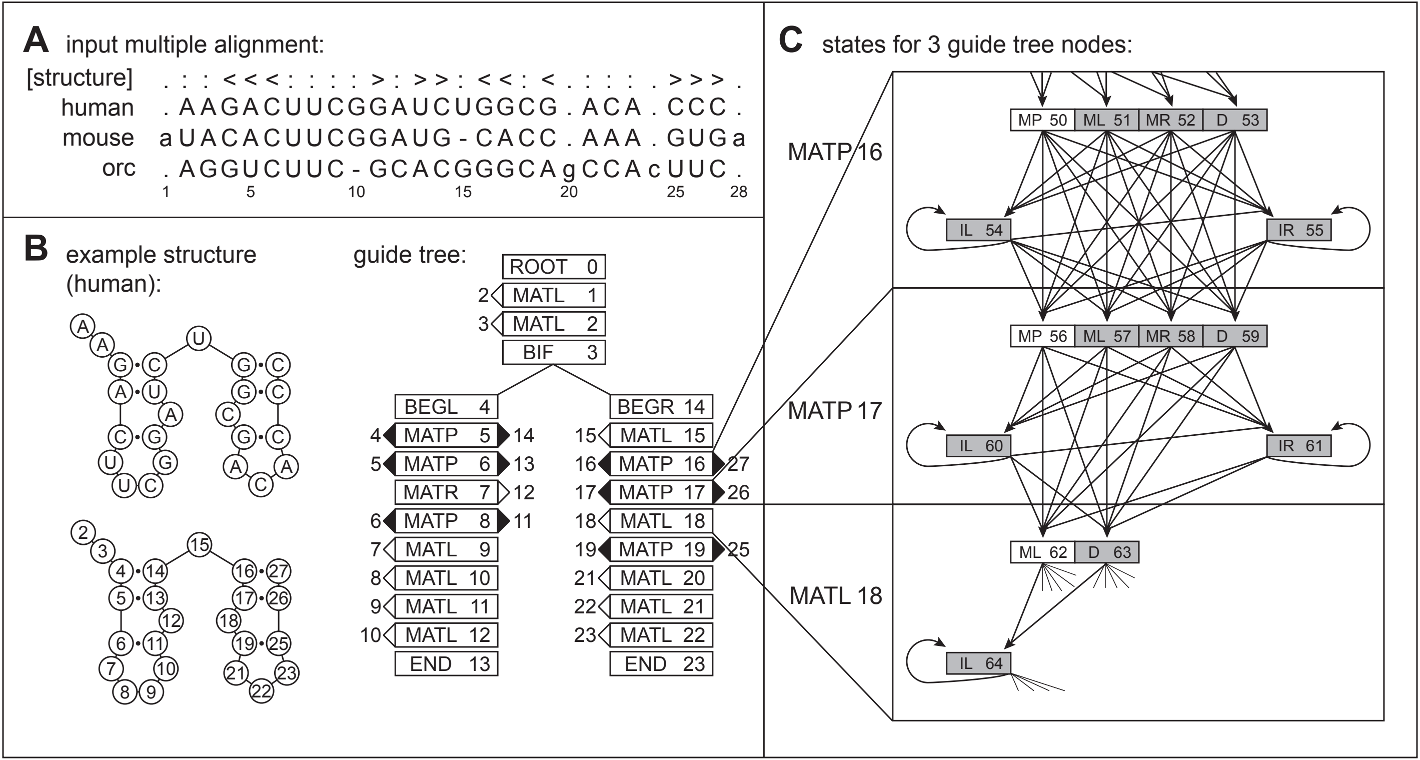
A input multiple alignment:
[structure] . : : < < < : : : : > : > > : < < : < . : : : . > > > .
human . A A G A C U U C G G A U C U G G C G . A C A . C C C .
mouse a U A C A C U U C G G A U G - C A C C . A A A . G U G a
orc . A G G U C U U C - G C A C G G G C A g C C A c U U C .
1 5 10 15 20 25 28
B example structure (human):
guide tree:
ROOT 0
MATL 1
MATL 2
BIF 3
BEGL 4
MATP 5
MATP 6
MATR 7
MATP 8
MATL 9
MATL 10
MATL 11
MATL 12
END 13
BEGR 14
MATL 15
MATP 16
MATP 17
MATL 18
MATP 19
MATL 20
MATL 21
MATL 22
END 23
C states for 3 guide tree nodes:
MATP 16
MATP 17
MATL 18
MP 50
ML 51
MR 52
D 53
IL 54
IR 55
MP 56
ML 57
MR 58
D 59
IL 60
IR 61
ML 62
D 63
IL 64
